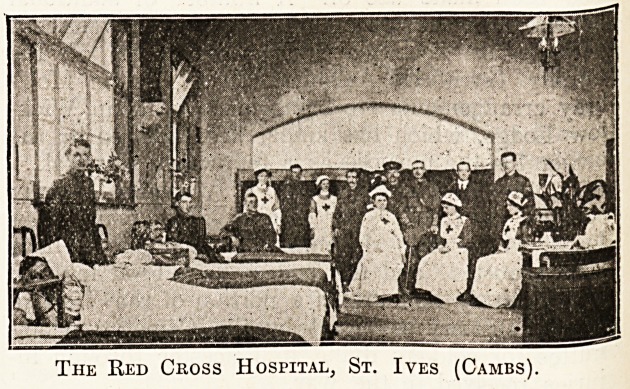# Hospitals and the War: Work for the Wounded of the Allied Armies

**Published:** 1914-11-14

**Authors:** 


### HOSPITALS AND THE WAR.

### Work for the Wounded of the Allied Armies.

### ST. GEORGE'S HOSPITAL.

A second batch of wounded from the Front were admitted into the hospital on Sunday, November 1, making forty-eight in all.

The cases were more severe than the previous ones, having all been engaged in the recent heavy fighting in the North of France.

A great many presents of game, fruit, games, tobacco, etc., have been received. A concert for the men was kindly arranged by the Misses Beringer to be held in the ward on Wednesday last.

### BARNSLEY: CASES FROM SHEFFIELD.

During the past fortnight the fifty beds allotted for the wounded at Beckett Hospital, Barnsley, have been occupied by cases transferred from the base hospital at Sheffield. Colonel Connell made an inspection on Thursday last week, the consequence of which resulted in thirty-one cases being reported as fit for convalescent homes. These were transferred to Sheffield, where they were distributed amongst various families in the district.

Previously, on October 26, thirty-seven wounded Belgian soldiers arrived at Beckett Hospital, being transferred from the base hospital, Sheffield. They were conveyed from the station by motor-cars to hospital, through the kindness of Messrs. Booker, Limited, and other friends. The wounded were met at the station by the Mayor and Mrs. M. Spenser-Stanhope; Mr. Pawsey, hon. secretary of the hospital; Dr. Trevor Howell, in charge of the St. John Ambulance Association; Acting-Major W. E. Raley, together with a company of ambulance men under the superintendence of Mr. Bellamy. On arriving at the hospital the wounded were received by the matron and the medical staff, Mr. G. A. Bond, Mr. A. Whitham, and Mr. H. Fensby. Musical parties and other friends are doing all that is necessary to cheer them up.

### BRISTOL.

On Sunday evening, October 25, 200 more British wounded arrived at Southmead Infirmary, Bristol. Many of the men had been in French hospitals before coming to England.

Another contingent of 162 arrived on the Monday following, consisting chiefly of those who had been fighting at Armentières—the Gordons, Rifle Brigade, Royal Field Artillery, and Cheshire Regiment. The men were taken to the Royal and Southmead Infirmaries. The Royal Army Medical Corps, in whose charge the wounded were sent, are reported to have said that the arrangements at Bristol were the best they had seen. A number of convalescents have recently been transferred from the Royal Infirmary in order to make room for the more serious cases. Ten who are Belgians have been sent to Cleve Hill, Downend, the residence of Sir Charles D. Cave, which has provision for fifty patients. Some twenty have been received at Corsham and Lydney Town Halls, which have been equipped by the local Red Cross Societies as temporary hospitals.

### CARDIFF: WOUNDED SENT DIRECT FROM FRANCE.

Another Red Cross train reached Cardiff just before midnight on November 2 in charge of Major Bird, of Penarth. It contained 151 wounded soldiers, twenty-six of whom were considered to be serious cases. The men came from the recent fighting in Belgium and the North of France, and though some of them had been treated at the base hospitals and remained there about ten days, others had been dispatched home with the utmost speed. In many instances their clothes were torn, muddy, and bloodstained. "We have had a particularly rough time," said one wounded man who came from Belgium, "fighting all the time we were there, and with no rest to speak of. Yes, we were quite equal to them in material, but there were so many thousands of the Germans that they seemed to be coming up fresh all the time." Not a few Welshmen were among them. The arrangements at the railway station were in charge of Colonel Hepburn and Major Ewan Maclean, with whom was Colonel East, whilst Commandant Sparrow and about 120 men of the Red Cross Society were in attendance and rendered most efficient service. Sixty of the wounded were taken to Howard Gardens School, thirty-five to Albany Road, forty to Splott Road, and sixteen to King Edward VII.'s Hospital.

### LEICESTER: THE 5th NORTHERN GENERAL HOSPITAL.

The great pressure which was reported at the Leicester Base Hospital in our last issue [the name of the Leicester Royal Infirmary was inadvertently inserted] has to some extent been relieved by the transfer of patients to other institutions. By an arrangement made by the commanding officer and the borough authorities, the isolation hospital on the Groby Road has been utilised for the reception of thirty of the less serious cases; other cases have been sent to the Desford Hall Convalescent Home, and other available homes have also been made use of. A number of additional homes for the reception of semi-convalescent patients has been placed at the disposal of the County Territorial Association. At Melton Mowbray arrangements are being made to fit up Wicklow Lodge, which has kindly been allotted by the owner for the purpose as a temporary hospital.

Of the last two convoys of wounded Belgian soldiers received at the base hospital, many were found to be seriously injured. Four deaths have been reported, and the bodies have been interred with military honours in a portion of the Welford Road Cemetery set apart by the Cemetery Committee for soldiers dying in Leicester through injuries received in the war.

The patients at the Leicester Royal Infirmary, sixty-two in number, are making good progress, but there are several serious cases. Some of the men are partially convalescent, and are able to take exercise in the grounds. The nursing of this large addition to the patients of the institution has been a heavy burden upon Miss Vincent, the matron, and her nursing staff, who have shown great readiness and have regarded it a privilege to lend assistance at a time of national stress.

### ANCOATS HOSPITAL: BELGIAN APPETITES.

An urgent appeal for beds having been received from the military authorities, the committee of Ancoats Hospital, Manchester, placed twenty beds at their disposal, and accordingly twenty Belgian soldiers are now patients of this hospital.

A few of these men had been defenders of Ghent, and when retiring marched from five o'clock in the morning until three o'clock in the afternoon without a break. Strong and well built, nearly all are under thirty, three being under twenty. Suffering, as most of them were, from bullet and shrapnel wounds, in many cases septic, the worst case was that of a gangrenous foot, which the surgeons have since had to remove. Their appetites seem enormous compared with those of ordinary English patients, and their request is always for more. They are also permitted to smoke. One of these patients speaks four languages—English, French, Flemish, and German—and acts as general interpreter for the others, though the hospital is fortunate in having Nurse Brassey, who is a French linguist.

Gifts of clothing are daily being received, though so far only two pairs of boots have come to hand, but as a member of the hospital committee has kindly offered to provide boots where necessary this difficulty has been overcome. Out of twenty patients received only two had one pair of boots each, and one had one boot only.

### MIDDLESBROUGH.

A further batch of fifty wounded soldiers, of whom ten were Belgians, arrived at Middlesbrough on October 26, *en route* for Hemlington Hospital, which stands a few miles out of the town. The soldiers were received at the railway station by the 29th Yorks Red Cross Detachment, and conveyed by a fleet of motors through the town to the hospital. The use of the North Riding Infirmary's horse ambulance was granted for the conveyance of a number of serious "stretcher" cases. The sixty beds which the authorities of the North Riding Infirmary placed at the disposal of the War Office for the reception of wounded have not yet been required for that purpose, but since the beginning of August the infirmary has regularly received the cases of sickness and accident arising amongst the troops at the Tees defences.

### SHEFFIELD: THE AMBULANCE PROBLEM.

Within the last week nearly 200 more wounded have arrived at the Sheffield Base Hospital in two batches. In the first batch there were more serious cases than have been previously sent to Sheffield. Some of the men arrived at Sheffield with signs of tetanus. The difficulties which the medical staff of the Northern General Hospital had to contend with in dealing with large numbers of "stretcher" cases arriving at Sheffield have now been almost overcome. The scarcity of ambulance appliances was very noticeable when nearly fifty "cot" cases arrived at the Midland Station. Mr. F. M. Tindall, who kindly took up this matter, made an appeal for more ambulances, which has met with a generous response. Sir Joseph Jones has had one of his motor-cars transformed into an up-to-date ambulance. Mr. Reuben Thompson has given up his Wolseley, and had it fitted up as a four-stretcher ambulance, having also provided four other stretchers so that four may be in use while four others are being loaded. Mr. Arthur Peech has handed over his private motor-car and fitted it up most elaborately as an ambulance for two "stretcher" cases with six sitting cases. Mr. W. N. Drew, of Newton, Chambers, and Co., and Mr. T. W. Ward, the ex-Master Cutler, have, with many others, followed this example, while Mr. Tindall with subscriptions which have been sent in by numerous people has been able to purchase two Fords for the use of the hospital. When the next batch of wounded arrive in Sheffield the hospital authorities will have fifteen ambulances at their disposal for their removal, apart from the use of the Sheffield Corporation omnibuses and ambulances.

### WAKEFIELD.

The first batch of wounded soldiers arrived at the Wakefield Clayton Hospital on Friday, November 6. They came from the 2nd Northern General Hospital, Leeds, the distributing centre for the northern part of the West Riding. There were thirty-one, and the V.A.D. carried out a smart piece of work, as they only heard at noon that they were to send over to Leeds to fetch them and by 2.30 ten motors were ready. The cases are not very bad, except four or five, most of them having received good attention at Leeds previously. Cars to fetch them were kindly lent by private owners, and when the soldiers arrived they were met at the hospital by Mr. Percy Tew (the president of the hospital), Dr. Walker (the surgeon in charge), Mr. Arthur H. Barnes (the secretary of the hospital), Lady Catherine Milnes Gaskell (the Vice-President of the V.A.D.), and Mrs. King (Commandant V.A.D., West Riding). Immediately on arrival the men were entertained to a good tea and were soon made comfortable. They all seem very cheerful, though they have evidently some of them had a bad time, as in two cases slight amputations were necessary, and one soldier had received twenty-two wounds from a Maxim. The auxiliary hospital at the Clayton Hospital consists of two wards which the Governors have placed at the disposal of the V.A.D., and as soon as the men are convalescent they will be moved on to convalescent homes, when a further batch will be sent.

### WORTHING: FIRST BATCH FROM THE FRONT.

Although some weeks ago convalescent wounded were received at Worthing, it was not until Oct. 27 that thirty patients were sent to Worthing Hospital direct from the Front. The men belonged to the Duke of Cornwall's Light Infantry, East Surrey, Gordons, Royal Horse Artillery, Royal Army Medical Corps, Rifle Brigade, Army Service Corps, and other regiments. Doctors Hinds, Hyde, Good, and Brown were in attendance at the station, together with the Voluntary Aid Detachments.

### ASHTON-UNDER-LYNE: ANTI-TYPHOID VACCINE.

The local infirmary received its first contingent of wounded soldiers on October 26, twenty-one men being transferred from the 2nd Western General Hospital, Manchester, in the afternoon of that day. A further draft of seven men has since been admitted. One of the first contingent, Corporal W. Black, 2nd Life Guards, received an intimation on November 4 that a commission had been granted him, and he has been the recipient of many congratulations and good wishes. The troops passing through the Ashton Barracks are attending the local infirmary in batches of fifty on two afternoons a week for treatment with anti-typhoid vaccine. Many soldiers who have been sent from the Territorial hospitals to their homes in the district are attending the out-patient department of the infirmary. In addition to the beds in the infirmary above referred to, there are in the military hospital near by twenty-five beds occupied by wounded soldiers. There are also twelve beds in use at the Mechanics' Institute, Ashton, and twenty-five at Early Bank, Stalybridge, the residence of Mr. Philip Talbot, F.R.C.S. There must be considerably over a hundred Belgian refugees housed in Ashton-under-Lyne, Stalybridge, and the immediate neighbourhood.

### BRISTOL: 400 BRITISH AND BELGIAN.

With but about an hour's notice of their coming some 250 Belgian wounded arrived at Bristol on October 31. Col. Bush, commanding the 2nd Southern Hospital, was present at the station. Surgical cases were sent to the Royal Infirmary and the remainder to Southmead. There were a few "stretcher" cases. Since then some 150 British wounded arrived, of whom about fifty were "stretcher" cases.

### ST. IVES (CAMBS) RED CROSS HOSPITAL.

A Red Cross hospital has been established at St. Ives by converting a large schoolroom with the offices adjacent into the requisite accommodation, which Colonel Griffiths, M.D., M.C., F.R.C.S., of the 1st Eastern General Hospital, has approved. The Fife and Forfar Yeomanry have been quartered at St. Ives, and the hospital has

The Red Cross Hospital, St. Ives (Cambs).

been most useful. All inoculations have been carried out at the hospital. This hospital and the Voluntary Aid Detachment were the first to be recognised by the War Office. The hospital is in charge of Mrs. Weston, with trained and Red Cross nurses, and has been inspected by Sir Ian Hamilton. Mrs. Weston (*née* Harrison) received her training at Addenbrooke's Hospital, Cambridge, where she afterwards held for some years the post of sister of the Women's Medical Ward.

**[Some accounts are unavoidably held over.]**

## Figures and Tables

**Figure f1:**